# Sex- and age-related differences in renal and cardiac injury and senescence in stroke-prone spontaneously hypertensive rats

**DOI:** 10.1186/s13293-023-00519-6

**Published:** 2023-05-22

**Authors:** Aneesa Ansari, Sarah L. Walton, Kate M. Denton

**Affiliations:** 1 Department of Physiology, Monash University, Melbourne, VIC Australia; 2 Cardiovascular Disease Program, Monash Biomedicine Discovery Institute, Monash University, Melbourne, VIC Australia

**Keywords:** Cell senescence, Kidney, Heart, Ageing, Sex differences, Stroke prone spontaneously hypertensive rats

## Abstract

**Background:**

Sex differences play a critical role in the incidence and severity of cardiovascular diseases, whereby men are at a higher risk of developing cardiovascular disease compared to age-matched premenopausal women. Marked sex differences at the cellular and tissue level may contribute to susceptibility to cardiovascular disease and end-organ damage. In this study, we have performed an in-depth histological analysis of sex differences in hypertensive cardiac and renal injury in middle-aged stroke-prone spontaneously hypertensive rats (SHRSPs) to determine the interaction between age, sex and cell senescence.

**Methods:**

Kidneys, hearts and urine samples were collected from 6.5- and 8-month-old (Mo) male and female SHRSPs. Urine samples were assayed for albumin and creatinine content. Kidneys and hearts were screened for a suite of cellular senescence markers (senescence-associated β-galactosidase, p16^INK4a^, p21, γH2AX). Renal and cardiac fibrosis was quantified using Masson’s trichrome staining, and glomerular hypertrophy and sclerosis were quantified using Periodic acid–Schiff staining.

**Results:**

Marked renal and cardiac fibrosis, concomitant with albuminuria, were evident in all SHRSPs. These sequelae were differentially affected by age, sex and organ. That is, the level of fibrosis was greater in the kidney than the heart, males had greater levels of fibrosis than females in both the heart and kidney, and even a 6-week increase in age resulted in greater levels of kidney fibrosis in males. The differences in kidney fibrosis were reflected by elevated levels of cellular senescence in the kidney in males but not females. Senescent cell burden was significantly less in cardiac tissue compared to renal tissue and was not affected by age or sex.

**Conclusions:**

Our study demonstrates a clear sex pattern in age-related progression of renal and cardiac fibrosis and cellular senescence in SHRSP rats. A 6-week time frame was associated with increased indices of cardiac and renal fibrosis and cellular senescence in male SHRSPs. Female SHRSP rats were protected from renal and cardiac damage compared to age-matched males. Thus, the SHRSP is an ideal model to investigate the effects of sex and aging on organ injury over a short timeframe.

**Supplementary Information:**

The online version contains supplementary material available at 10.1186/s13293-023-00519-6.

## Plain Language Summary

Kidney and cardiovascular diseases are some of the leading causes of death worldwide, and they affect men and women differently. Young men are generally at higher risk of developing these diseases than young women. Women also have unique risk factors for kidney and cardiovascular disease. These may include complications associated with pregnancy, such as preeclampsia, and menopause. For example, the risk of disease for women increases significantly after menopause. In addition, treatment strategies for kidney and cardiovascular diseases are often less effective in women compared to men, but the causes for this are unknown. More research is needed to understand sex differences in kidney and cardiovascular diseases, so that we can develop new drugs that are effective in women as well as men. In this study, we have examined kidney and heart damage associated with elevated blood pressure in adult male rats and adult female rats (long before the onset of menopause). We have shown that males develop significantly more scarring of their hearts and kidneys compared to females. We also identified the cells in the kidneys of male rats, but not female rats, showed signs of DNA damage and early ageing. This suggests cellular damage in young males may contribute to their more rapid progression of kidney disease compared to females. Future research examining females after menopause, when disease risk is greater, will enhance our understanding of cell damage in kidney and cardiovascular disease.

**Supplementary Information:**

The online version contains supplementary material available at 10.1186/s13293-023-00519-6.

## Highlights

- Male stroke-prone spontaneously hypertensive rats presented with albuminuria and histological indices of kidney and cardiac injury at 6.5 months of age, and these sequelae were exacerbated over a 6-week timeframe.
- Females exhibited a degree of protection against kidney and cardiac damage with respect to age-matched males.
- The cellular senescence burden was greater in kidneys compared to hearts of male animals, despite both organs displaying widespread fibrosis.
- The interaction between sex and cellular senescence in the development of cardiovascular and kidney diseases warrants further investigation.

**Supplementary Information:**

The online version contains supplementary material available at 10.1186/s13293-023-00519-6.

## Introduction

Hypertension is the most common chronic disease across the globe and represents a major risk factor for developing renal and cardiovascular diseases [1]. Epidemiological studies demonstrate sex differences play a critical role in the incidence and severity of hypertension [2–5]. Men are at a higher risk of developing hypertension and experience accelerated progression of chronic renal and cardiovascular diseases than age-matched premenopausal women [6, 7]. After menopause, the risk of hypertension and associated organ damage in women increases significantly [4]. Marked sex differences at the cellular and tissue level may contribute to susceptibility to hypertension and end-organ damage.

Accumulation of senescent cells in cardiovascular tissues occurs in response to stressors, such as hypertension [5, 8]. Senescent cells acquire a senescence-associated secretory phenotype (SASP), releasing a mixture of inflammatory factors such as interleukin (IL)-6 and IL-8 [9, 10] that contribute to cardio-renal injury. The SASP causes neighboring cells to undergo senescence, promotes fibroblast proliferation and extracellular matrix accumulation and induces cytokine activation to recruit immune cells, including macrophages and T-cells [9, 10]. Interestingly, age-related sex differences influence the accumulation of senescent cells in cardiovascular tissue. For example, mice heterozygous for ERCC1-XPF, a DNA repair endonuclease required for DNA repair, show a marked enhancement of age-related cell senescence across a wide range of tissues including aorta and kidney [11]. This effect was greater in males, at least until close to the end of life, when levels increased in females to match males [11]. In ERCC1-XPF heterozygous mice, cell senescence was highest in the aorta and greater in males than females. This is in agreement with previous studies showing endothelial cells are particularly susceptible to senescence [11]. Another study, in Wistar rats, showed that renal expression of p53 and p21, levels of oxidative stress and telomere shortening were greater in 15Mo than 3Mo old male Wistar rats, but not in age matched females [9]. In humans, leucocyte telomere length was shorter in men than women between 30 and 70 years of age, but shortened more rapidly in women towards ageing [12]. Furthermore, arteries from postmenopausal women showed telomere uncapping was 2.5-fold greater than premenopausal women [13]. Thus, sex differences in cell senescent burden exist across the lifespan, but how this interacts with chronic hypertension remains to be elucidated.

Here, we aimed to examine whether sex- and age-related differences in renal and cardiac injury and cellular senescence are evident in stroke-prone spontaneously hypertensive rats (SHRSPs). Marked sex- and age-related differences in blood pressure and kidney and cardiac damage [14–17] have been well-characterized in the SPSHR, and the susceptibility to hypertensive kidney disease is accounted for by two known foci [18]. We hypothesized that sex- and aged-related renal and cardiac injury would differentially affect markers of cell senescence in the SHRSPs. To achieve this aim, we examined indices of cardiac and renal damage in conjunction with a suite of cellular senescence markers in renal and cardiac tissue in middle-aged male and female SHRSP rats. As kidney and cardiovascular disease rapidly progresses in the SHRSP, we examined whether changes in cellular senescence were detectable over a 6-week time frame.

## Methods

### Animals

Experiments were conducted in accordance with the Australian Code of Practice for the Care and Use of Animals for Scientific Purposes and approved by the Monash University School of Biomedical Sciences Animal Ethics Committee. The SHRSP, a model of severe progressive hypertension and advanced renal damage, was used to investigate sex differences in cardiac/renal injury and senescence. SHRSPs at 6.5 (male: *n* = 10; female: *n* = 8) and 8 (male: *n* = 10; female: *n* = 11) months of age (Mo) were obtained from the Monash Animal Research Precinct, Monash University (VIC, Australia). Male and female WKY rats were obtained from the Animal Resources Centre (WA, Australia) at 3Mo of age (*n* = 3/group) and function to highlight widespread tissue injury evident in SHRSPs. All rats were housed under standard conditions with temperature maintained at 24–26 °C and a 12-h light–dark cycle. Rats had ad libitum access to standard rodent chow (0.35% sodium, 20% crude protein, 8.5% crude fat and 3.2% crude fibre; cat. #102119, Barastoc, Ridley Agricultural Products, WA, Australia) and water.

### Post-mortem tissue collection and urinalysis

Rats were euthanized via carbon dioxide inhalation and blood collection via cardiac puncture. Bladder puncture was performed to collect urine samples. Hearts and kidneys were dissected. Transverse midline sections of tissue were fixed in 4% paraformaldehyde (PFA) for processing to paraffin, or in a commercially available fixative (1% glutaraldehyde, 5% methanol in formaldehyde; #9860, CST, Massachusetts, USA) designed for SA-β-gal staining, a marker of cellular senescence. Urinary albumin and creatinine levels were determined using commercially available mouse kits (Nephrat and Creatinine Companion, Exocell, Philadelphia, USA).

### Histology

Heart and kidney samples fixed in 4% PFA were processed to paraffin and sectioned at 4 μm. Representative midline sections from each kidney and heart were stained with Periodic acid–Schiff (PAS) and Masson’s Trichrome by Monash Histology Platform. The Aperio Scanscope AT Turbo scanner (Leica Microsystems, NSW, Australia) was used to generate digital images of the sections (20 × magnification). All analyses were performed by a researcher blinded to treatment groups using Aperio ImageScope software (Leica, RRID:SCR_020993), Fiji ImageJ (RRID:SCR_002285) or QuPath version 0.2.3 (RRID:SCR_018257) [19].

#### Kidney morphometry

Glomerular area in PAS-stained kidney sections was quantified by tracing glomerular borders when the vascular pole was evident. Thirty glomeruli were analysed per rat and measurements averaged. PAS-positive staining within the selected glomeruli was quantified using the Positive Pixel Count Algorithm optimised for PAS staining within the Aperio ImageScope software. The number of tubular casts in five randomly selected fields of view (1.5 mm^2^) were counted.

#### Interstitial fibrosis

The renal cortex and cardiac left ventricle were delineated, and collagen was detected using the Positive Pixel Count Algorithm optimised for Masson’s trichrome staining. Fibrosis was expressed as a percentage of positively stained pixels to total pixels within the renal cortex or cardiac left ventricle.

#### Immunohistochemical detection of cellular senescence markers

Paraffin slides were dewaxed through 3 × 2 min changes of xylene and rehydrated in washes of ethanol (3 × 2 min of 100% ethanol) and distilled water. Slides were subjected to antigen retrieval in citrate buffer (pH 6.0) at 98 °C for 20 min. Endogenous peroxidase activity was blocked by incubation with 0.3% H_2_O_2_ in distilled water for 20 min. Non-specific binding was blocked in 10% goat serum in antibody diluent (Cat. No. S0809, DAKO, CA, USA) for 1 h at room temperature. Slides were incubated with primary antibodies (Table 1; one slide per animal per antibody) in a sealed chamber overnight at 4 °C. Slides were thoroughly washed and then incubated with the relevant biotinylated secondary antibodies (1:200 dilution; Vector Laboratories, CA, USA). Slides were washed, treated with avidin/biotin complex (Elite ABC Kit, Vector Laboratories, CA, USA) for 30 min and the reaction developed with diaminobenzidine (DAB, Vector Laboratories, CA, USA). Sections were counterstained with haematoxylin, coverslipped and scanned using the Aperio Scanscope AT Turbo scanner (Leica Microsystems, NSW, Australia). Five randomly selected fields of view (FOV; × 200; 1.5 mm^2^) were extracted per animal and visualized using QuPath version 0.2.3 (RRID:SCR_018257) [19]. For morphological quantification, the number of γH2AX positive (+), p21 + and p16^INK4a+^ cells were counted manually in each FOV.

**Table 1:** Antibodies

Antibodies	Species	Dilution	Manufacturer
Primary antibody			
yH2AX	Rabbit	1:500	Cell Signaling Technology Cat# 9718, RRID:AB_2118009
p16^INK4a^	Rabbit	1:100	Abcam Cat# ab211542, RRID:AB_2891084
p21	Rabbit	1:100	Cell Signaling Technology Cat# 2947, RRID:AB_823586
Secondary antibody			
Anti-rabbit, biotinylated		1:200	Vector Laboratories Cat# BA-1000, RRID:AB_2313606

#### Senescence-associated β-galactosidase staining

To prepare tissues for SA-β-gal staining, samples were cryoprotected post-fixation in 30% sucrose overnight, frozen in OCT and sectioned at 4 µm. Sections were incubated in SA-β-gal staining solution overnight at 37 °C and counterstained with nuclear fast red. The proportion of heart and kidney tissue positive for SA-β-gal staining was quantified using Aperio ImageScope software.

### Statistical analysis

Data are represented as the mean ± standard error mean (SEM). Statistical analysis was performed using Graph Pad Prism version 9 (GraphPad software Inc., CA, USA). Data were tested for normality using a Shapiro–Wilk test. Data that did not pass the normality test (ACR, tubular casts, and several cellular senescence markers within the kidney: p21, p16^INK4a^, yH2AX) were analysed via a Kruskal-Wallis test followed by Dunn’s multiple comparisons. All other data were determined to fit a Gaussian distribution. For these data, SHRSP groups were analysed via two-way ANOVA with the factors age (*P*_age_; 6 Mo or 8.5 Mo), sex (*P*_sex_; male or female) or glomerular area (P_area_) and their interaction, followed by post hoc Sidak’s tests for multi-group comparisons as appropriate. Data for WKY male and female rats were analysed using Student’s *t*-test. Linear regression analysis was used to determine correlations between albuminuria, fibrosis and senescence markers in the SHRSPs (Table 2). Two-sided *P* ≤ 0.05 was considered statistically significant.

**Table 2:** Correlation analyses of albuminuria and renal fibrosis/cellular senescence markers in male SHRSPs

	Fibrosis	SA-β-gal	p16^INK4a^	p21	γH2AX
ACR	*r*^2^ = 0.26 ***P* = 0.03**	*r*^2^ = 0.20 *P* = 0.06	*r*^2^ = 0.58 ***P* = 0.0002**	*r*^2^ = 0.44 ***P* = 0.003**	*r*^2^ = 0.03 *P* = 0.46
Fibrosis		*r*^2^ = 0.03 *P* = 0.45	*r*^2^ = 0.09 *P* = 0.19	*r*^2^ = 0.03 *P* = 0.50	*r*^2^ = 0.04 *P* = 0.38
SA-β-gal			*r*^2^ = 0.09 *P* = 0.21	*r*^2^ = 0.04 *P* = 0.42	*r*^2^ = 0.008 *P* = 0.91
p16^INK4a^				*r*^2^ = 0.68 ***P* < 0.0001**	*r*^2^ = 0.04 *P* = 0.41
p21					*r*^2^ = 0.12 *P* = 0.14

Bold values indicate statistical significance. No correlations were detected in female SHRSPs. *ACR* urinary albumin-to-creatinine ratio

## Results

### Albuminuria and glomerular injury were greater in male compared to female SHRSP

**Fig. 1:**
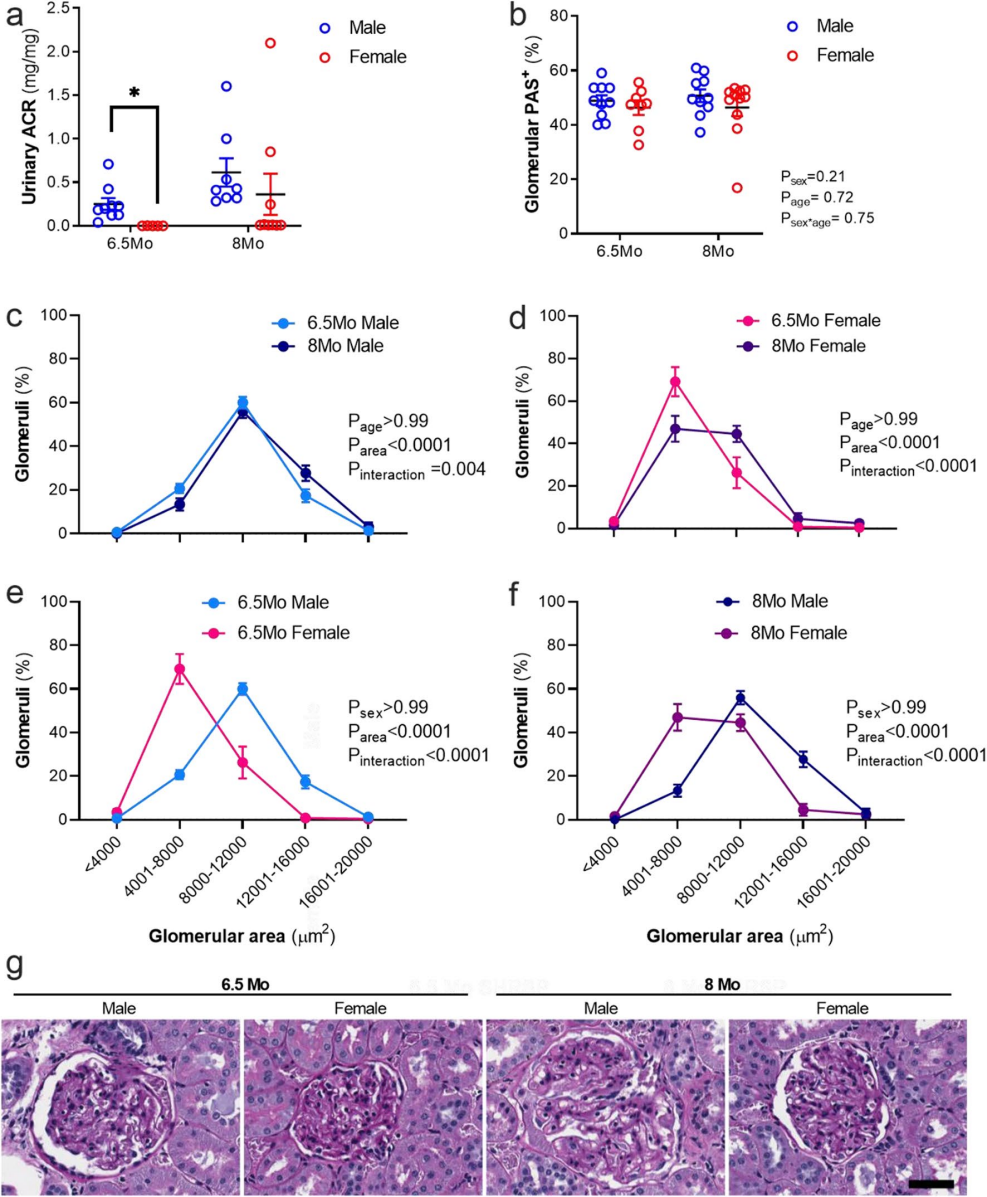
Albuminuria and glomerular morphology in male and female SHRSP rats. **a** Urinary albumin:creatinine ratio (ACR); *n* = 5–9/group). **b** Quantification of glomerulosclerosis (mean glomerular % PAS-staining). **c**–**f** Glomerular size distribution, an index of glomerular hypertrophy, presented as the percentage of glomeruli vs glomerular area (µm^2^); *n* = 8–11/group. **g** Representative images of periodic acid-Schiff (PAS) staining; scale = 50 µM for all images. Data shown as mean ± SEM. **a** Data analyzed via a Kruskal–Wallis test with Dunn’s multiple comparisons (*P < 0.05). **b** Data via two-way ANOVA. **c**–**f** Groups analysed via two-way ANOVA followed by Tukey’s multiple comparisons tests. *P*_interaction_ indicates a shift in the relationship between group comparisons

**Fig. 2:**
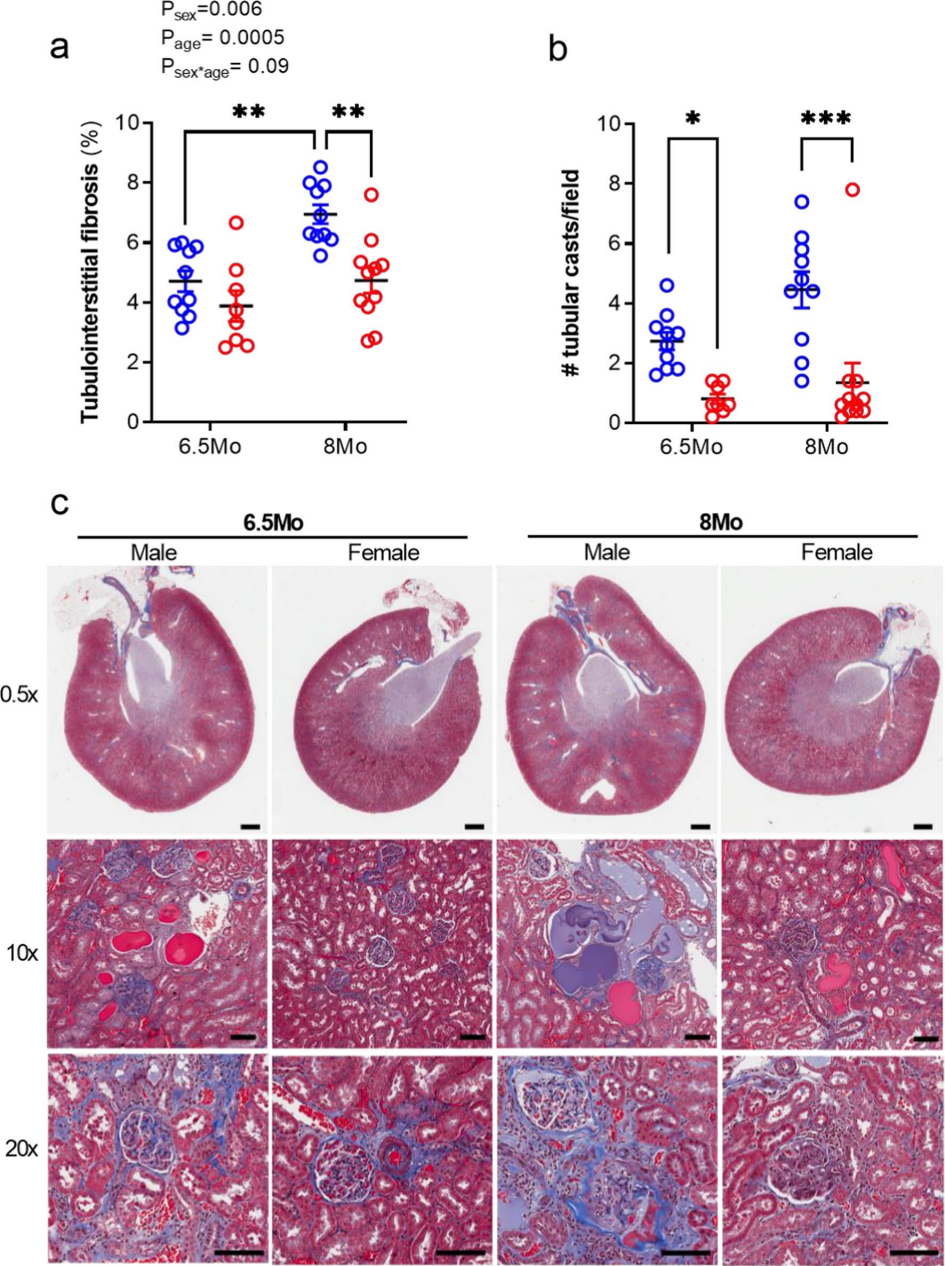
Kidney tubulointerstitial fibrosis and tubular casts in male and female SHRSP rats. **a** Tubulointerstitial fibrosis (% collagen) within the renal cortex. **b** Mean number of tubular casts present per field of view (1.5 mm^2^). **c** Representative images of Masson’s trichrome staining showing collagen deposition (stained blue) and tubular casts. 0.5 × magnification: scale = 1 mm. 10 × and 20 × magnification: scale = 50 µm. Values represent mean ± SEM; *N* = 8–11/group. **a** Data analysed by two-way ANOVA with Sidak’s multiple comparison test; **P < 0.01. **b** *p < 0.05 and ***p < 0.001 via a Kruskal–Wallis test with Dunn’s multiple comparisons

The urinary albumin-to-creatinine ratio (ACR) in 6.5 Mo male SHRSPs was significantly greater than 6.5 Mo female SHRSPs (*P* < 0.05, Fig. 1a). No statistical significance was detected between the sexes at 8 Mo (Fig. 1a). By contrast, albuminuria was not present in male or female WKYs (Additional file 1: Fig. S1a). Marked glomerulosclerosis was observed in all SHRSPs (Fig. 1g) but not WKYs (Additional file 1: Fig. S1b, j). Glomerular PAS^+^ staining, as a marker of glomerulosclerosis, was not affected by age or sex in the SHRSP male and female rats (*P*_sex_ = 0.21, *P*_age_ = 0.72; Fig. 1b, g). Glomerular surface area distribution was shifted rightward in male compared to female WKYs and SHRSPs, indicating that males of both strains had larger glomeruli (all *P*_interaction_ < 0.0001; Fig. 1e, f, Additional file 1: Fig. S1c). Glomerular area in SHRSPs ranged between ~ 3700 and 20,000 µm^2^ (Fig. 1c–d). There was a ~ 10% shift in glomerular surface area distribution between 6.5 Mo and 8 Mo in male SHRSP (*P*_interaction_ = 0.004; Fig. 1c). Older female SHRSPs had larger glomeruli than 6.5Mo female SHRSPs (*P*_interaction_ < 0.0001; Fig. 1d). For example, female SHRSPs had ~ 69% at 6.5 Mo of age and ~ 45% at 8 Mo age of glomeruli in the range of ~ 4001–8000 µm^2^ (*P*_interaction_ < 0.0001; Fig. 2d).

### Kidney fibrosis and tubular damage was exacerbated by age in male SHRSPs

Masson’s trichrome staining revealed widespread, moderate to severe tubulointerstitial fibrosis in kidneys from all SHRSP groups (Fig. 2a, c) but not WKYs (Additional file 1: Fig. S1d, j). Tubulointerstitial fibrosis was greater in 8 Mo vs 6.5 Mo SHRSP males (*p* < 0.01), but no age difference was found in females (Fig. 2a). Renal tubulointerstitial fibrosis was greater in 8Mo male SHRSP compared to similarly aged females (*p* < 0.001; Fig. 2a). Thus, there was a sex (*P*_sex_ = 0.006) and age effect on tubulointerstitial fibrosis (*P*_age_ = 0.0005; Fig. 2a) in SHRSP, but there was only a trend for the effect of age to be different between the sexes (*P*_sex*age_ = 0.09). Tubular casts were largely absent in WKY animals (Supplemental Fig. 1e) and female SHRSPs (Fig. 2b, c). However, tubular casts were abundant in the renal cortex of male SHRSPs at both ages (both *p* < 0.05 vs age-matched females; Fig. 2b), with a trend towards greater numbers of casts in the 8Mo cohort (*p* = 0.06 vs 6.5 Mo males).

### Senescent cell burden in the kidney was greater in male SHRSPs

**Fig. 3:**
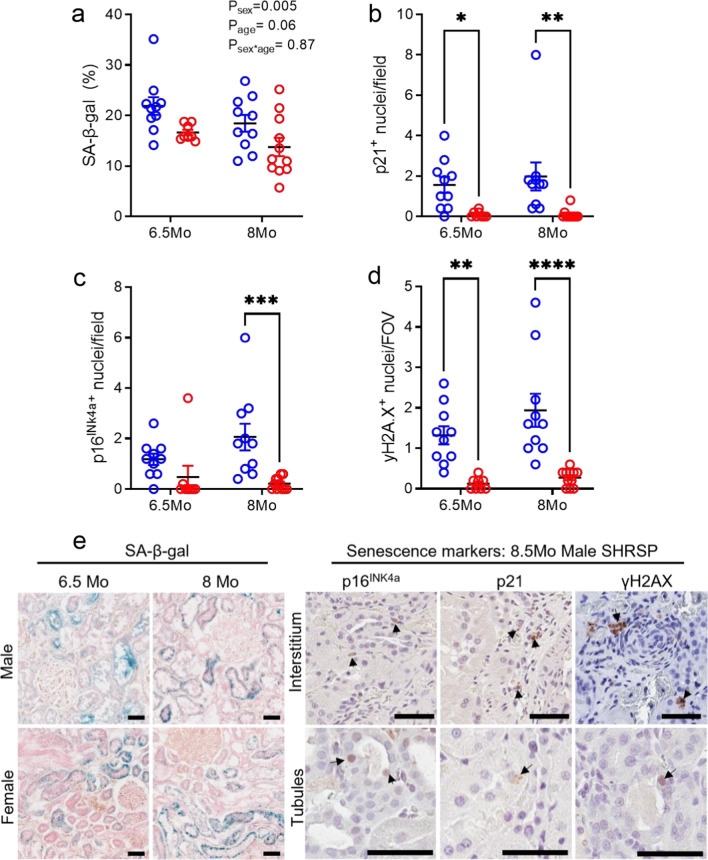
Cellular senescence markers in kidneys of male and female SHRSP rats. **a** Senescence-associated β-galactosidase (SA-β-gal) activity within the renal cortex (% positive staining). Cells per field of view (FOV; 1.5 mm^2^) positive (+) for (**b**) p21, (**C**) p16^INK4a^ and (**d**) **γ**H2AX immunostaining. **e** Representative images of SA-β-gal activity in kidneys from all groups. p16^INK4a^, p21 and **γ**H2AX immunostaining in the kidney interstitium and tubular cells of 8.5 Mo male SHRSP rats. Scale = 50 µm for all images. Values represent mean ± SEM; *N* = 8–11/group. **a** Data analysed via two-way ANOVA with a Sidak’s post hoc test. **b**–**d** *p < 0.05, **p < 0.01, ***p < 0.001 and ****p < 0.0001 via a Kruskal–Wallis test with Dunn’s multiple comparisons

A suite of cellular senescence markers was used to determine the localization and abundance of senescent cells within renal tissue [20]. We first screened the kidney samples for SA-β-gal activity, a surrogate marker of enhanced lysosomal biogenesis [20, 21]. SA-β-gal activity in the renal cortex was minimal in all WKYs (Additional file 1: Fig. S1f, j) but high in all SHRSPs (Fig. 3a, e). In the SHRSPs, SA-β-gal was predominantly localised to the proximal and distal tubules in the renal cortex (Fig. 3e). The proportion of SA-β-gal positive tissue within the renal cortex was greater in male than female SHRSP at both 6.5 Mo and 8 Mo (*P*_sex_ = 0.005, Fig. 3a).

We next examined the localization and expression of the cyclin-dependent kinase inhibitors, p21 and p16^INK4a^, as indicators of irreversible exit from the cell cycle. Neither marker were detected in WKY kidneys (Additional file 1: Fig. S1g, i). In contrast, p21 and p16^INK4a^ immunostaining was present in nuclei of cortical tubules and tubulointerstitium in SHRSPs (Fig. 3b, c, e). Quantification revealed p21 expression was great in 6.5 Mo males compared to 6.5 Mo females (*p* < 0.05, Fig. 3b), but no difference in p16^INK4a^ expression was detected at this age (Fig. 3c). At 8Mo, both p21and p16^INK4a^ expression was greater in male SHRSPs compared to females (both *p* < 0.01; Fig. 3b, c, e). γH2AX, a selective marker of DNA double-strand breaks, followed the same trend, whereby expression was largely absent in WKYs (Additional file 1: Fig. S1h) and female SHRSPs (Fig. 3d), but significantly higher in the renal cortex of male SHRSPs (*p* < 0.01; Fig. 3d, e). Localization of γH2AX in SHRSPs was primarily within the tubulointerstitium and cortical tubule nuclei (Fig. 3e).

### Cardiac fibrosis was greater in male SHRSPs

**Fig. 4:**
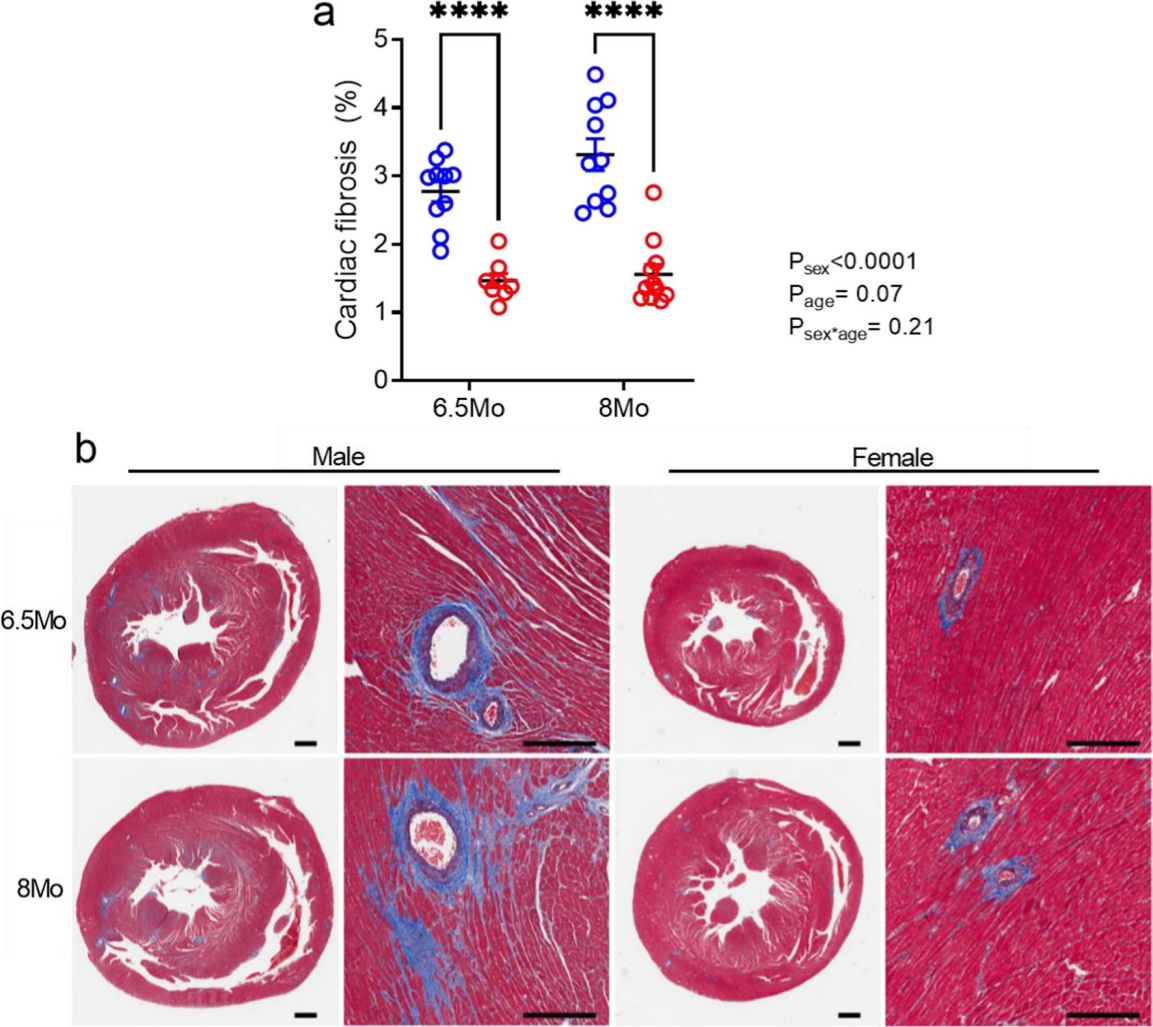
Cardiac fibrosis in male and female SHRSP rats. **a** Cardiac interstitial fibrosis (% positive staining). **b** Representative images of Masson’s trichrome staining showing collagen deposition (stained blue). Whole-heart cross sections: scale = 1 mm. Magnified images: scale = 400 µm. Values represent mean ± SEM; *N* = 8–11/group. ****p < 0.001 via two-way ANOVA with Sidak’s multiple comparison test

Masson’s trichrome staining showed widespread interstitial fibrosis in cardiac tissue from all SHRSPs (Fig. 4a, b) but not WKYs (Additional file 1: Fig. S2a). Cardiac fibrosis was significantly greater in male than female SHRSPs (6.5 Mo: *p* < 0.0001; 8 Mo: *p* < 0.0001; Fig. 4a), and in male than female WKYs (*p* = 0.03, Additional file 2: Fig. S2a). There was a trend for cardiac fibrosis to increase with age in the SHRSP (*P*_age_ = 0.07; Fig. 4a).

### Cardiac cellular senescence was not affected by age or sex in SHRSPs

**Fig. 5:**
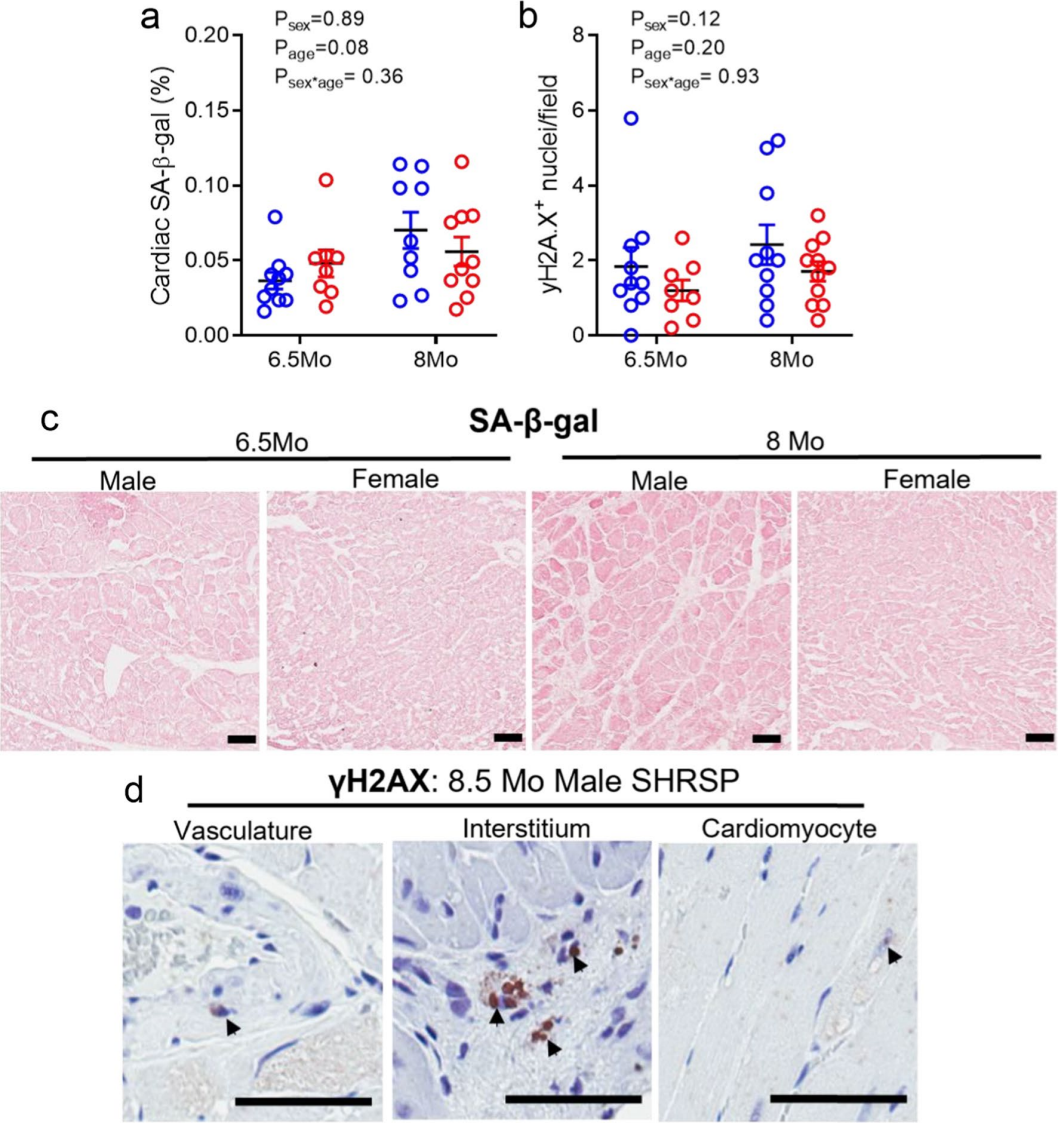
Cellular senescence markers in hearts of male and female SHRSP rats. **a** Senescence-associated β-galactosidase (SA-β-gal) activity within the heart (% positive staining). **b** **γ**H2AX positive (+) cells per field of view (FOV; 1.5 mm^2^). **c** Representative images of SA-β-gal activity in hearts from all groups. Scale = 50 µm. **d** **γ**H2AX immunostaining in the vasculature, interstitium and cardiomyocytes in hearts collected from 8.5 Mo male SHRSP rats. Scale = 50 µm. Values represent mean ± SEM; *N* = 8–11/group. Data analysed via two-way ANOVA

In contrast to the kidney, SA-β-gal staining was very low in cardiac tissue in all SHRSP groups (Fig. 5a, c) and WKYs (Additional file 2: Fig. S2b). p16^INK4a^ and p21 were not detected in cardiac tissue of either WKY or SHRSP animals. However, γH2AX expression was detected in cardiac tissue of SHRSPs (Fig. 5b, d) but not WKYs (Additional file 2: Fig. S2c). Specifically, γH2AX^+^ nuclei were localized to the vascular smooth muscle, interstitium and occasional cardiomyocytes (Fig. 5d). Expression was not affected by sex or age in the SHRSP (Fig. 5b).

### Associations between albuminuria, fibrosis and cellular senescence

We examined whether albuminuria in the SHRSP was associated with renal fibrosis and cellular senescence. Results from correlation analyses are presented in Tabl﻿e 2. ACR was correlated with renal fibrosis in male SHRSPs. Furthermore, there was a significant association in male SHRSPs between ACR and the accumulation of p16^INK4a+^ and p21^+^ cells within kidney tissue. Similarly, there was a trend towards an association between ACR and renal SA-β-gal activity (*p* = 0.06) in male SHRSPs. **γ**H2AX was not correlated with ACR/kidney injury markers in male SHRSPs. No associations were detected in female SHRSPs. 

## Discussion

Here we demonstrate marked organ fibrosis and senescent cell burden in the SHRSP is differentially affected by age, sex and organ. That is, the level of fibrosis was greater in the kidney than the heart, males had greater levels of fibrosis than females in both the heart and kidney, and a 6-week increase in age resulted in greater levels of organ fibrosis, particularly in males. These differences in kidney fibrosis were reflected by elevated levels of cellular senescence in the kidney, particularly in males. Therefore, the SHRSP presents as a useful tool to study the sex- and age-related progression of hypertensive organ damage.

Selective inbreeding of SHRs with the highest blood pressures resulted in the derivation of the SHRSP sub-strain [22], with a region on chromosome 17 identified as the contributor to higher blood pressure in the SHRSP [18, 23]. The SHRSP develops severe progressive hypertension from 4–5 weeks of age, with systolic blood pressure plateauing by 10–12 weeks of age at ~ 180 mmHg in males [24], and ~ 160 mmHg in females [16, 22]. SHRSP are salt sensitive and if fed a high salt diet, > 95% of SHRSPs die of stroke between 6 and 12 Mo [16, 22]. However, previous reports indicate SHRSPs, in the absence of salt loading, survive past 9Mo of age for males [25, 26], and past 15Mo of age for females [27]. In the present study, none of the male or female SHRSPs (of which all were fed a normal sodium diet) showed signs of stroke or died spontaneously by the final timepoint of 8.5Mo of age.

Hypertension is a potent driver of organ damage including fibrosis, glomerulosclerosis and proteinuria [5, 14]. As expected, we found evidence of progressive glomerular hypertrophy, albuminuria and tubular damage in male SHRSPs. In our study, glomerular size was greater in males than females. These findings are consistent with previous reports in rodents [28] and humans, where glomerular diameter (~ 20%) and volume (~ 34%) are greater in men than women [29]. This suggests that glomerular hypertrophy is delayed in females compared to males and may relate to lower blood pressure [16], although we acknowledge lack of blood pressure measurement as a limitation of this study. Glomerular hypertrophy reduces podocyte density, which drives podocyte foot process degeneration, mesangial matrix expansion, Bowman’s capsule thickening and ultimately glomerular scarring and loss of function [30, 31]. In the healthy kidney, little albumin is excreted [32]. However, with glomerular hypertrophy and podocyte injury, albumin filtration increases. The absolute level and rate of rise over time of albuminuria is a strong risk factor for chronic kidney disease progression [33, 34]. Albuminuria in the SHRSP increased with age in males not females, indicating that renal damage progressed over the 6-week period in males and conversely that females are protected against glomerular injury. The abundance of tubular casts, which are associated with proteinuric tubular damage [35], in the renal cortex of male SHRSPs mirrored the pattern of albuminuria in our animals. Therefore, in the SHRSP, susceptibility to glomerular hypertrophy and albuminuria is sex- and age-dependent.

Previous reports show male SHRSPs present with renal fibrosis from 3 months of age [16, 22]. In contrast, the SHR, with lower blood pressure than the SHRSP, does not develop renal tissue injury until > 6 Mo of age [36]. By 12Mo, in both male and female SHRSPs, renal and cardiac fibrosis is widespread [27, 37]. We found both male and female SHRSPs developed marked fibrosis in the kidney and heart by 6.5 Mo age. Interestingly, this injury was intensified within an age gap of 1.5 Mo, more so in male than female SHRSP. This finding shows that what may be considered a minor age difference may elicit significant differences in organ injury. An alternative viewpoint is the 6-week timeframe for a male SHRSP, for example, may represent ~ 15% of the total lifespan [25, 26]. This is a significant proportion of a lifespan to examine disease progression, highlighting the SHRSP as a useful preclinical model of rapid ageing. Another advantage of the SHRSP is that the females enter reproductive senescence earlier than SHR; 15Mo in SHRSP vs 18Mo in SHR [27]. Oestrogen provides a protective role against organ damage in females until menopause [5, 38] by downregulating fibroblast proliferation and collagen production, and preventing oxidative stress and senescence-associated inflammation [39–41]. Thus, in the future, the impact of age and loss of ovarian hormones could be examined over a shortened timeframe in the SHRSPs compared to other rat strains. Finally, the SHRSP has a strong genetic susceptibility to hypertensive kidney disease [18, 23], with different foci contributing to blood pressure elevation, glomerular injury/fibrosis, and proteinuria [18]. In light of this, the SHRSP is a valuable genetic model of hypertension in which the effects of sex and aging on different aspects of hypertensive kidney injury can be examined.

A relatively low proportion of senescent cells (~ 10%) in tissues can cause organ dysfunction and fibrosis [9, 42]. This leads to a vicious cycle as fibrosis develops with accelerating accumulation of senescent cells in the kidney [43, 44] and the heart [45] and associated release of the profibrotic, proinflammatory secretome. Cells exposed to stressors, such as oxidative stress, hypoxia or inflammation, are all factors associated with hypertension entering cellular senescence [8]. This type of senescence does not typically involve telomere shortening but still activates DNA damage response pathways [46]. Identifying senescent cells within organs represents several challenges, primarily due to the significant heterogeneity of subtypes and the lack of a 'universal’ senescent marker [10, 47]. To address this challenge, we measured the lysosomal signature of SA-β-gal in conjunction with the expression of ‘core’ senescence-associated proteins [47]. Marked SA-β-gal activity was detected in all SHRSP kidneys, but to a greater extent in males than females [48]. High baseline SA-β-gal activity may imply enhanced lysosomal biogenesis, and, therefore, autophagy, in the kidney of the SHRSP [10, 48]. Increases in lysosomal number and size is a common feature of senescent cells both in vitro and in vivo*,* and lysosomal dysfunction is considered a key contributor to the ageing and injury-repair processes [10]. Concomitant with elevated SA-β-gal activity, we showed two key pathways that lead to cell cycle arrest were enhanced in kidneys from male SHRSPs: (1) phosphorylated histone H2AX (**γ**H2AX) levels, an indicator of DNA damage [49] and (2) p21 and p16^INK4a^, cyclin-dependent kinase inhibitors. In chronic hypertension, DNA damage is thought to arise secondary to the generation of excess reactive oxygen species, as previously shown in rodent models of angiotensin II-induced and deoxycorticosterone acetate (DOCA)-salt-induced hypertension [50–52]. Therefore, DNA damage in kidneys and hearts of male SHRSPs may be the result of hypertension-associated oxidative stress. Upregulation of p21 expression is often detected in the early, rather than at the established phase of cellular senescence, and may reflect DNA damage [47]. p16^INK4a^ expression is activated later than p21, often following persistent stress signals [47, 53], For example, renal p16^INK4a^ expression is observed in patients with essential hypertension [54] and also experimental models of hypertension [10, 55]. Taken together, the suite of markers selected for this study indicate the acquisition of a senescence-associated phenotype within the renal tubules and tubulointerstitium in the male SHRSP. We hypothesis that hypertension in the SHRSP from an early age drives renal senescence burden and contributes to the progressive tubular degeneration and fibrosis. An alternate possibility is that a low senescence burden from a young age drives the hypertensive phenotype, as studies have shown as little as 10% senescent burden can elicit organ injury [56]. This could be tested via an ontology of senescence, oxidative stress and blood pressure prior to, and at the point of, hypertension arising. In SHRSPs, this would require examination of very young (< 10 weeks) animals. Finally, the associations detected between albuminuria and p16^INK4a^ and p21 expression in the male SHRSP suggests these cellular senescence markers may be useful prognostic markers and/or therapeutic targets for hypertensive kidney damage.

The lack of renal DNA damage and p21/p16^INK4a^ expression in female SHRSPs may reflect the protective effects of estrogen [41]. At the ages studied (up to 8Mo), estrous cycling is likely intact as the SHRSP strain enters reproductive senescence by 13–15 months of age, although vaginal cytology is required to confirm this. Estrogen appears to modulate senescence in several tissues. For example, ovariectomy in mice induces SA-β-gal accumulation in aortas [58] and also femur osteocytes [59], effects that were largely rescued by estradiol supplementation. The role of estrogen in regulating renal senescence has not been directly characterized, but should be addressed by examining the trajectory of cellular senescence, kidney function and blood pressure from young to reproductively senescent SHRSPs. We hypothesize estrogen plays a protective effect against renal senescence in hypertension, and may contribute to the extended lifespan of female compared to male SHRSPs. An alternate possibility is there are innate differences in cell cycle regulation between the sexes, independent of sex hormones. This represents a knowledge gap that must be addressed, while senolytics to treat chronic diseases are under development.

Interestingly, we found the kidneys were more prone to accumulation of senescent cells than the hearts of the SHRSP. The lack of SA-β-gal, p21 and p16^INK4a^ expression in cardiac tissue implies this pathway, at least in middle-aged SHRSPs, is not a key contributor to cardiac fibrosis. However, we observed elevation in the DNA damage marker **γ**H2AX, indicating a degree of DNA damage is a feature of hypertensive cardiac injury in both sexes. Organ-specific differences in senescence cell burden have previously been shown in mice, where age-related increases in p16^INK4a^ and p21 were not detected in heart tissue [11]. This points to a degree of renal hypersensitivity to hypertensive stimuli, protective mechanisms against cellular senescence in the heart, and/or organ-specific biases to either apoptotic or senescent pathways. A senescence phenotype, however, may play beneficial and detrimental roles in diseased states. Beneficial effects of acute cellular senescence are seen in several contexts, such as wound healing and cancer, where a shift towards a senescence phenotype promotes clearance of damaged cells and prevents their proliferation [10, 20, 53]. Thus, an alternate possibility is that the presence of senescent cells in the SHRSP kidney may represent a renoprotective mechanism against hypertension-induced injury.

Sex differences in cellular senescence is a burgeoning field. Further research is necessary to understand the pathways via which biological sex influences cellular senescence, with consideration for cell and organ type. Future studies may wish to explore the beneficial vs detrimental effects of cellular senescence in hypertension using senolytic agents to clear senescent cells. A key focus should be placed on sex differences to ensure efficacy and safety of senolytics in both sexes. We acknowledge important limitations of the present study. First, we have not examined the time course of organ injury and senescent cell burden in the WKY, which precludes age-matched comparisons between the WKY and SHRSP. However, WKYs are a well-characterised control for the SHRSP, and do not show any signs of renal disease until much later in life. For example, the male WKY can live for > 90 weeks without showing any evidence of proteinuria [60]. Second, while we hypothesize the renal injury and senescence is driven by blood pressure elevation, we have not measured blood pressure in this study. Measurement of blood pressure in animals via the gold-standard technique of radiotelemetry would allow us to determine whether blood pressure is the primary driver of renal injury and senescence. Finally, future studies may seek to interrogate the interaction between cellular senescence and sex hormones via gonadectomy and/or hormonal replacement.

### Perspectives and significance

Our study demonstrates a clear sex pattern in age-related progression of renal and cardiac fibrosis and cellular senescence in SHRSP rats. Kidneys and hearts from all SHRSPs exhibited widespread fibrosis and injury. Senescent cell burden was significantly higher in kidney compared to cardiac tissue. Progressive fibrosis in kidneys of male rats was detected over just a 1.5-month timespan. Female SHRSPs were relatively protected from renal and cardiac injury compared to their male counterparts. Therefore, biological sex of the SHRSP has significant ramifications for the progression of heart and kidney injury, and senescent cell accumulation. We conclude the SHRSPs are a useful model to study the complex relationships between ageing, sex and hypertensive organ damage.

## Supplementary Information

**Additional file 1: Figure S1.** Albumin excretion, kidney pathology and cellular senescence in young WKY rats. **a** Urinary albumin: creatinine ratio; **b** Quantification of glomerulosclerosis. **c** Glomerular size distribution, an index of glomerular hypertrophy, presented as the percentage of glomeruli vs glomerular area. **d** Tubulointerstitial fibrosiswithin the renal cortex. **e** Mean number of tubular casts present per field of view. **f** Senescence-associated β-galactosidase activity within the renal cortex. Cells per field of view positive for **g** p16^INK4a^, **h** **γ**H2AX and **i** p21 immunostaining. Data shown as mean ± SEM; n = 3/group. **a,b,d–i** Data analyzed by an unpaired Student’s t-test. **c** Groups analysed via two-way ANOVA followed by Tukey’s multiple comparisons tests. P_interaction_ indicates a shift in the relationship between group comparisons. **j** Representative images of Masson’s trichrome staining showing collagen deposition; 0.5 × magnification: scale = 1 mm. 10 × and 20 × magnification: scale = 50 µm. Representative images of SA-β-gal activity in kidneys; scale = 50 µM. Representative images of glomerular PAS staining; scale = 50 µM.

**Additional file 2: Figure S2.** Cardiac fibrosis and cellular senescence in young WKY rats. **a** Fibrosis within cardiac tissue. **b** Senescence-associated β-galactosidase activity within the renal cortex. **c** Cells per field of view positive for **γ**H2AX. Data shown as mean ± SEM; n = 3/group. Data analyzed by an unpaired Student’s t-test. **d** Representative images of Masson’s trichrome staining showing collagen deposition. Whole-heart Masson’s trichrome cross section: scale = 1 mm; high-power Masson’s trichrome image: scale = 400 µm. Representative images of SA-β-gal activity in heart tissue; scale = 50 µM.

## Data Availability

The data sets used and/or analysed during the current study are available from the corresponding author on reasonable request.
