# Supplementary figures and images for: Sex- and age-related differences in renal and cardiac injury and senescence in stroke-prone spontaneously hypertensive rats
Source: Biol Sex Differ. 2023 May 22;14:33. doi: 10.1186/s13293-023-00519-6 (PMC10201739; doi:10.1186/s13293-023-00519-6)

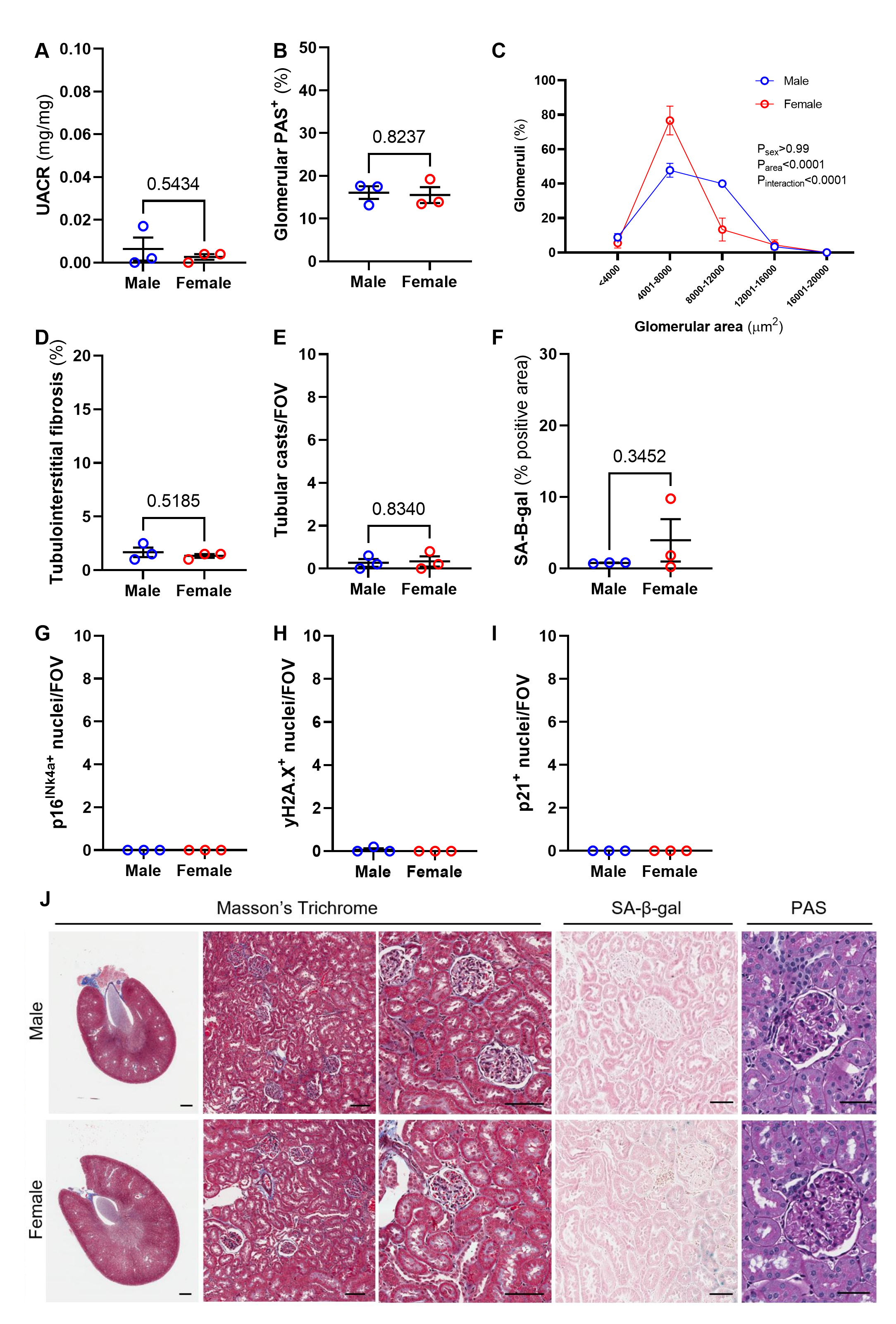

Supplement: Supplementary file 1 — Additional file 1: Figure S1. Albumin excretion, kidney pathology and cellular senescence in young WKY rats. a Urinary albumin: creatinine ratio; b Quantification of glomerulosclerosis. c Glomerular size distribution, an index of glomerular hypertrophy, presented as the percentage of glomeruli vs glomerular area. d Tubulointerstitial fibrosiswithin the renal cortex. e Mean number of tubular casts present per field of view. f Senescence-associated β-galactosidase activity within the renal cortex. Cells per field of view positive for g p16INK4a, h γH2AX and i p21 immunostaining. Data shown as mean ± SEM; n = 3/group. a,b,d–i Data analyzed by an unpaired Student’s t-test. c Groups analysed via two-way ANOVA followed by Tukey’s multiple comparisons tests. Pinteraction indicates a shift in the relationship between group comparisons. j Representative images of Masson’s trichrome staining showing collagen deposition; 0.5 × magnification: scale = 1 mm. 10 × and 20 × magnification: scale = 50 µm. Representative images of SA-β-gal activity in kidneys; scale = 50 µM. Representative images of glomerular PAS staining; scale = 50 µM. [file 13293_2023_519_MOESM1_ESM.tif]

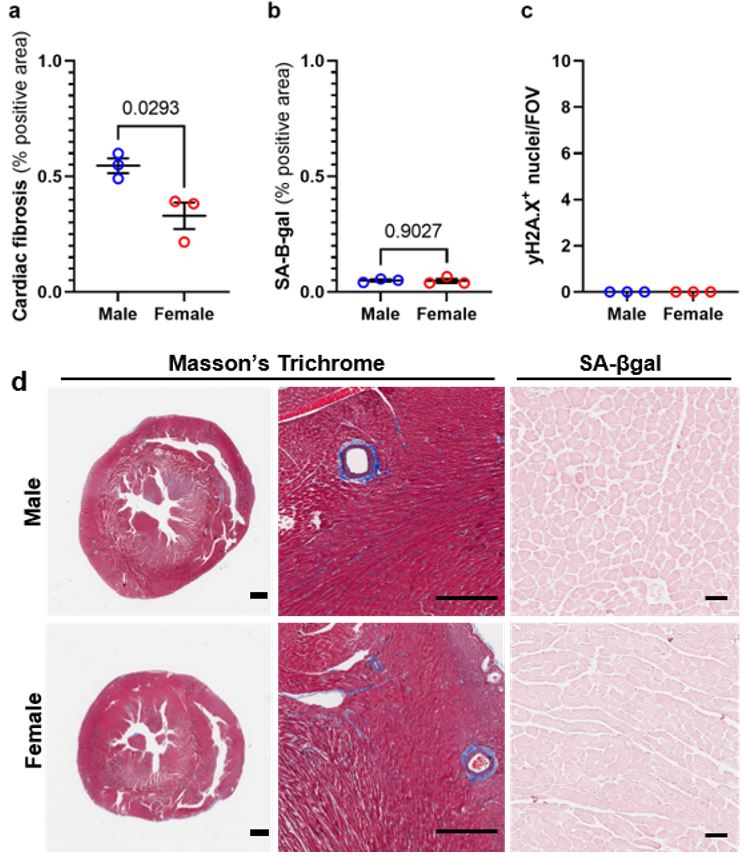

Supplement: Supplementary file 2 — Additional file 2: Figure S2. Cardiac fibrosis and cellular senescence in young WKY rats. a Fibrosis within cardiac tissue. b Senescence-associated β-galactosidase activity within the renal cortex. c Cells per field of view positive for γH2AX. Data shown as mean ± SEM; n = 3/group. Data analyzed by an unpaired Student’s t-test. d Representative images of Masson’s trichrome staining showing collagen deposition. Whole-heart Masson’s trichrome cross section: scale = 1 mm; high-power Masson’s trichrome image: scale = 400 µm. Representative images of SA-β-gal activity in heart tissue; scale = 50 µM. [file 13293_2023_519_MOESM2_ESM.jpg]
